# Supplementary material for: The Role of Prophage ϕSa3 in the Adaption of Staphylococcus aureus ST398 Sublineages from Human to Animal Hosts
Source: Antibiotics (Basel). 2024 Jan 23;13(2):112. doi: 10.3390/antibiotics13020112 (PMC10886223; doi:10.3390/antibiotics13020112)
Supplement: Supplementary file 1 [file antibiotics-13-00112-s001.zip › antibiotics-2814879-supplementary.pdf]

Supplementary Table 1. Clinical and molecular summary of all ST398 isolates in our study.

Isolate	Country (City)	Year	Host	Age	Additional information	Case	Source	Clade	MRSA/ MSSA	SCC*mec*	PVL	*spa* type
GD1095	China (Guangzhou)	2010	Human	Child	Community	Colonization	Nasal	I	MSSA	-	-	t571
GD1098	China (Guangzhou)	2010	Human	Child	Community	Colonization	Nasal	I	MSSA	-	-	t571
GD1042	China (Guangzhou)	2010	Human	Child	Community	Colonization	Nasal	IIGOI	MSSA	-	-	t034
GD1088	China (Guangzhou)	2010	Human	Child	Community	Colonization	Nasal	IIGOI	MSSA	-	-	t571
GD1413	China (Guangzhou)	2010	Human	Child	Community	Colonization	Nasal	IIGOI	MSSA	-	-	t034
GD1539	China (Guangzhou)	2010	Human	Child	Community	Colonization	Nasal	IIGOI	MSSA	-	+	t571
GD2002	China (Guangzhou)	2010	Human	Child	Community	Colonization	Nasal	IIGOI	MSSA	-	-	t034
GD930	China (Guangzhou)	2010	Human	Child	Community	Colonization	Nasal	IIGOI	MSSA	-	-	t571
GD1703	China (Guangzhou)	2010	Human	Child	Community	Colonization	Nasal	IIGOI	MSSA	-	-	t034
387T	Canada (Calgary)	2014	Human	Adult	Clinical (CUPS)	Colonization	Throat	I	MSSA	-	-	t571
387N	Canada (Calgary)	2014	Human	Adult	Clinical (CUPS)	Colonization	Nasal	I	MSSA	-	-	t571
215N	Canada (Calgary)	2014	Human	Adult	Clinical (STI)	Colonization	Nasal	I	MSSA	-	-	t571
215W	Canada (Calgary)	2014	Human	Adult	Clinical (STI)	Infection	Wound	I	MSSA	-	-	t571
293G	Canada (Calgary)	2014	Human	Adult	Clinical (STI)	Colonization	Groin	I	MSSA	-	-	t1451
232N	Canada (Calgary)	2014	Human	Adult	Clinical (STI)	Colonization	Nasal	I	MSSA	-	-	t571
GD53.1	China (Guangzhou)	2010	Human	8	Hospital	Colonization	Nasal	IIGOI	MSSA	-	-	t034
GD1067	China (Guangzhou)	2010	Human	Child	Community	Colonization	Nasal	IIGOI	MSSA	-	-	t571
GD1414	China (Guangzhou)	2010	Human	Child	Community	Colonization	Nasal	IIGOI	MSSA	-	-	t571
GD1449	China (Guangzhou)	2010	Human	Child	Community	Colonization	Nasal	IIGOI	MSSA	-	-	t1451
GD1211	China (Guangzhou)	2010	Human	Child	Community	Colonization	Nasal	I	MSSA	-	-	t571
GD149	China (Guangzhou)	2010	Human	13	Community	Colonization	Nasal	IIGOI	MSSA	-	-	t034
GD1259	China (Guangzhou)	2010	Human	Child	Community	Colonization	Nasal	I	MSSA	-	-	t571
GD1930	China (Guangzhou)	2010	Human	Child	Community	Colonization	Nasal	IIGOI	MSSA	-	-	t034
GD5	China (Guangzhou)	2010	Human	8	Hospital	Colonization	Nasal	IIGOI	MRSA	V	-	t034
GD1616	China (Guangzhou)	2010	Human	Child	Community	Colonization	Nasal	IIGOI	MSSA	-	-	t571
GD1853	China (Guangzhou)	2010	Human	Child	Community	Colonization	Nasal	IIGOI	MSSA	-	-	t571
GD104	China (Guangzhou)	2010	Human	13	Hospital	Colonization	Nasal	IIGOI	MSSA	-	-	t571
GD33	China (Guangzhou)	2010	Human	8	Hospital	Colonization	Nasal	IIGOI	MSSA	-	-	t1451
GD2000	China (Guangzhou)	2010	Human	Child	Community	Colonization	Nasal	IIGOI	MSSA	-	-	t034
GD223	China (Guangzhou)	2010	Human	Child	Community	Colonization	Nasal	IIGOI	MSSA	-	-	t034
08S-0030	Canada (Winnipeg)	2008	Human	Adult	Clinical	na	na	IIa1i	MRSA	V	-	t034
CF-EHF	Canada (Calgary)	2005	Human	19	Clinical (CF)	Chronic infection	Sputum	IIa1i	MSSA	-	-	t034

GD1696	China (Guangzhou)	2010	Human	Child	Community	Colonization	Nasal	IIGOI	MSSA	-	-	t034
GD1706	China (Guangzhou)	2010	Human	Child	Community	Colonization	Nasal	IIGOI	MSSA	-	-	t034
GD1677	China (Guangzhou)	2010	Human	Child	Community	Colonization	Nasal	IIGOI	MSSA	-	-	t034
GD1884	China (Guangzhou)	2010	Human	Child	Community	Colonization	Nasal	IIGOI	MSSA	-	-	t034
GD1108	China (Guangzhou)	2010	Human	Child	Community	Colonization	Nasal	I	MSSA	-	-	t571
GD1130	China (Guangzhou)	2010	Human	Child	Community	Colonization	Nasal	I	MSSA	-	-	t571
GD1517	China (Guangzhou)	2010	Human	Child	Community	Colonization	Nasal	IIGOI	MSSA	-	-	t034
GD1428	China (Guangzhou)	2010	Human	Child	Community	Colonization	Nasal	IIGOI	MSSA	-	+	t1451
GD705	China (Guangzhou)	2010	Human	13	Community	Colonization	Nasal	IIGOI	MSSA	-	-	t011
GD399	China (Guangzhou)	2010	Human	12	Community	Colonization	Nasal	IIGOI	MSSA	-	-	t034
GD487	China (Guangzhou)	2010	Human	11	Community	Colonization	Nasal	I	MSSA	-	-	t034
GD1025	China (Guangzhou)	2010	Human	Child	Community	Colonization	Nasal	IIGOI	MSSA	-	-	t034
N09-00266	Canada (Winnipeg)	2009	Human	Adult	Clinical	na	na	IIa	MRSA	V	-	t1250
